# Epigenetic role of the nuclear factor NF-Y on *ID* gene family in endometrial tissues of women with endometriosis: a case control study

**DOI:** 10.1186/s12958-019-0476-9

**Published:** 2019-03-15

**Authors:** Shirin Amirteimouri, Manan Ashini, Fariba Ramazanali, Reza Aflatoonian, Parvaneh Afsharian, Maryam Shahhoseini

**Affiliations:** 1 Department of Basic Sciences and Advanced Technologies in biology, University of Science and Culture, Tehran, Iran; 2 Reproductive Epidemiology Research Center, Royan Institute for Reproductive Biomedicine, ACECR, P.O. Box: 19395-4644, Tehran, Iran; 3 Department of Genetics, Reproductive Biomedicine Research Center, Royan Institute for Reproductive Biomedicine, ACECR, P.O. Box: 19395-4644, Tehran, Iran; 4 Department of Endocrinology and Female Infertility, Reproductive Biomedicine Research Center, Royan Institute for Reproductive Biomedicine, ACECR, Tehran, Iran

**Keywords:** Nuclear transcription factor Y, *ID* gene family, Endometriosis, Epigenetic

## Abstract

**Background:**

A predominant difference between endometrial and normal cells is higher proliferation rate in the former cells which is benign. The genes of inhibitor of differentiation (*ID*) family play a major role in cell proliferation regulation which might be targeted by the nuclear transcription factor Y (NF-Y) for subsequent epigenetic modifications through the CCAAT box regulatory region. The present study was designed to investigate the epigenetic role of NF-Y on *ID* gene family in endometrial tissue of patients with endometriosis.

**Materials & methods:**

In this case-control study, 20 patients with endometriosis and 20 normal women were examined for the relative expression of the *NF-YA*, *NF-YB*, *NF-YC* and *ID* genes by real-time PCR during the proliferative phase. The occupancy of NF-Y on CCAAT box region of *ID* genes was investigated using chromatin immunoprecipitation (ChIP) followed by real-time PCR.

**Results:**

The *NF-YA* was over-expressed in eutopic endometrium during the proliferative phase. Although the expression level of *NF-YB* and *NF-YC* were unchanged in eutopic samples, they were remarkably higher in ectopic group (*P*<0.05). The *ID2* and *ID3* genes were up-regulated in ectopic and eutopic tissues, however *ID1* and *ID4* genes were down-regulated in these samples (*P*<0.05). The ChIP analysis revealed significant enrichment of NF-Y on regulatory regions of *ID2,3* genes in eutopic group, but reduced binding level of NF-Y to the *ID1,3* promoters in ectopic specimens (*P*<0.05).

**Conclusion:**

The ability of NF-Y to regulate *ID* genes via CCAAT box region suggests the possible role of NF-Y transcription factor in epigenetic changes in endometrial tissues which may open novel avenues in finding new therapeutic strategies.

## Background

Endometriosis is an estrogen-dependent inflammatory disease that is strongly associated with infertility and affects over 70 million women worldwide [1, 2]. Although there is no single theory of pathogenesis to entirely explain all the manifestations of endometriosis [3], Sampson’s theory of retrograde dissemination of menstrual debris has gained widespread acceptance as an explanation for the initiating steps in the pathogenesis of endometriosis [4]. There are several leading theories, including altered immunity, coelomic metaplasia, and metastatic spread attempting to explain the origin of endometriosis. Recent studies have also found genetic basis for endometriosis [5]. None of these theories fully explain the whole mechanisms associated with the development of disease and the actual cause remains unknown [6, 7]. Multiple factors including genetics, epigenetics, environmental modifications, aging, and diverse anatomical or biochemical aberrations of uterine function are also involved in the development of endometriosis [8, 9].Accumulating evidence suggest that various epigenetic aberrations may play a significant role in the initiation and progression of endometriosis [10]. Epigenetic modifications such as DNA methylation, chromatin modifications, and RNA interference refer to heritable changes in gene expression with no underlying alteration in the genetic sequence [11].

Nuclear transcription factor Y (NF-Y) is one of the transcriptional regulation factors which mediates the above-mentioned epigenetic modifications [12]. NF-Y complexes are trimeric proteins composed of NF-YA, NF-YB, and NF-YC subunits that play a crucial role in regulating eukaryotic gene expression. A heterodimer of NF-YB and NF-YC which are homologous in sequence to histonesH2B and H2A, respectively, interacts with NF-YA to form the heterotrimeric NF-Y complex [13]. All of these subunits are necessary for binding to the core CCAAT box, a cis element present in 30% of eukaryotic promoters [14, 15]. Among the various *DNA binding proteins* that *interact* with this sequence, *NF-Y* is the only protein which needs all the CCAAT box *nucleotides to be able to bind the DNA and has an extremely specific interaction with this region* [16]*.* NF-Y has been shown to (i) mediate the recruitment of polymerase II onto various CCAAT box-containing promoters to permit the transcriptional activation (a pioneering role in activation of transcription) [17], (ii) induce DNA compaction that facilitates promoter-enhancer interactions, and (iii) regulate several cell cycle regulatory genes which are known to be critical for expression control [18, 19]. Although, it has been proved that the NF-Y complex is involved in critical biological processes including cell growth, proliferation/apoptosis balance, tumorigenesis (the elevated levels of NF-Y is associated with breast [20], ovarian [21], prostate [22], and thyroid [23] cancers), and cell reaction to the stressors, the exact roles of NF-Y on regulatory regions of many developmental genes remain poorly understood [21, 24].

The inhibitor of differentiation (*ID*) gene family consisting of *ID-1*, *ID-2*, *ID-3*, and *ID-4* isoforms are known to be dominant negative regulators of differentiation, but the positive regulators of cellular proliferation [25]. The ID helix-loop-helix (HLH) proteins lack the basic DNA binding region and are functional inhibitors of the basic helix-loop-helix (bHLH) transcription factors [26]. Recent data reported non-canonical functions for ID proteins, such as binding to Rb family and biochemical attributes including regulating cell fate, proliferation, differentiation, and migration [27, 28].

Since the exact roles of NF-Y on regulatory regions of *ID* genes are not still clarified, the current investigation was designed to evaluate the possible epigenetic role of NF-Y on *ID* gene family through CCAAT box region in endometrial tissue of endometriosis and non-endometriosis women.

## Material & methods

### Patients and tissue collection

Twenty patients with endometriosis (in stages III and IV), who underwent laparoscopic excision of ectopic endometrium lesions (ovarian endometrioma) were recruited for this study. The patients ranged in age from 20 to 45 years and had regular menstrual cycles. The exclusion criteria for participation were: endometrial hyperplasia, benign masses like fibroids and polyps, inflammatory diseases, autoimmune diseases, endocrine diseases, cancers, sepsis, asthma, glomerulonephritis, osteoporosis, psoriasis, myocardial infarction, and leukemia. It should be mentioned that based on the entity of the study, all analyzed samples were representing whole tissues, which are composed of a mixed population of cells (stromal, epithelial, and inflammatory).

The eutopic endometrium samples from these patients were obtained by pipelle. All tissue samples, i.e. both eutopic (20 samples) and ectopic (20 samples) endometrium, were collected, immediately divided into two separate cryovials (one contained RNAlater (QIAGEN Ltd) for RNA extraction and the other was used in ChIP assays), and stored at − 80 °C until performing the analysis. All biopsies were performed by an experienced gynecologist at the Royan Institute for Reproductive Biomedicine.

The control group consisted of 20 women with no evidence of endometriosis and having at least one child by natural pregnancy. Endometrium samples were taken from normal women during diagnostic laparoscopy. Freshly recovered tissues also divided into two separate cryovials (one contained RNAlater (QIAGEN Ltd) for RNA extraction and the other was used in ChIP assays), and stored at − 80 °C until performing the analysis. The exclusion criteria for the women from the control group were the same as for the patients. Before sampling, the nature of the study was explained and written informed consent was obtained from all participants.

### RNA isolation

Each tissue sample (50 mg approximately) preserved in RNALater was homogenized using scalpel blade and glass homogenizer. Total RNA was extracted with TRIzol reagent (Invitrogen, USA) according to the manufacturer’s instructions. Diethylpyrocarbonate (DEPC)-treated water was used for the dilution of the RNA pellet. The approximate concentration and purity of RNA was assessed by optical density [29] 260/280 ratios. To eliminate genomic DNA contamination from RNA samples, DNase I digestion was performed.

### Reverse transcription (RT) and real-time polymerase chain reaction analysis

Individual RNA samples from ectopic, eutopic, and control tissue samples (*n* = 60) were reverse transcribed into complementary DNA (cDNA). Standard RT was carried out in reaction mixture containing 4 μLof 5X RT Buffer, 2 μL of mixed dNTPs (2 .5μm each), 1 μL of RT-MULV enzyme, 1 μL Random hexamer, 1 μL RNase Inhibitor and 1 μg RNA template. The mixture was incubated in 25 °C for 5 min, 42 °C for 60 min and 70 °C for 5 min. To study the relative mRNA expression of the *NF-YA*, *NF-YB* and *NF-YC* genes, real-time PCR was carried out using Step One Plus™ Real-time PCR System (Applied Biosystems International, Inc., Switzerland). Every target cDNA was co-amplified withglyceraldehyde-3-phosphate dehydrogenase (GAPDH) as endogenous control using specific primers. Real-time PCR was performed in triplicate for each sample according to the following conditions: 5 μL of SYBR Premix Ex TaqII (Takara, Japan), 1 μL of each primer (5 pmol/μL), 11 μL H_2_O and 2 μL cDNA (12.5 ng/μL) in a final volume of 20 μL for each reaction. The reaction conditions were 95 °C for 4 min, followed by 40 cycles at 95 °C for 10 s, 60 °C for 30 s and 72 °C for 30s and final extension of 72 °C for 10 min. The relative mRNA expression was calculated for each sample according to the 2^−ΔΔ*C*T^and*GAPDH*gene was used as normalizer for the 2^−ΔΔ*C*T^calculus.All primer sets used in qRT-PCR are shown in Table 1.

**Table 1:** Primer pairs used in this study

Genes	Primer Sequences (5′-3′)	Product length (bp)
*NF-YA*	F: TTCTCCAGCAAGTTACAGTC	183
	R: ACCATCATGACCATCCCT	
*NF-YB*	F: TGCCATCAAGAGAAACGG	151
	R: ACTGCTCCACCAATTCC	
*NF-YC*	F: AGTATATCCGCTTAGCCCA	96
	R: TCTGTCTGTGTAATCTGTTGAG	
*GAPDH*	F: CTCATTTCCTGGTATGACAACGA	122
	R: CTTCCTCTTGTGCTCTTGCT	
*ID1*	F: CCTTGCTGTTCTGAAACCC	193
	R: GTGGAATGAGAGTGCGGA	
*ID2*	F:TGATAGACGTGCCACCTTCC	103
	R: TCAGAATGAAGCCCGAGCC	
*ID3*	F:CACAAGATAATTCCTGACGCCA	204
	R: AGTCCGCCTTTAGCCCAA	
*ID4*	F:CGCACGGCTCTATAAATACA	160
	R:GTGTCCTAGTCACTCCCTT	

### Chromatin immunoprecipitation-real-time PCR analysis

For ChIP assays, homogenized endometrial tissues were cross-linked with 1% formaldehyde and subjected to immunoprecipitation after sonication. The ChIP experiments were performed using the Orange ChIP kit (Diagenode, Belgium), according to the manufacturer’s instructions on three biological replicates. Real time PCR was used to amplify specific promoter DNA (*ID1–4*) bound to the immunoprecipitated histones (NF-Y) after reversing the histone-DNA cross-links (primers listed in Table 1). All the samples were amplified in triplicate. Data were represented as the percentage of input DNA associated with immunoprecipitated NF-Y relative to input chromatin. A Rabbit polyclonal antibody against NF-Y (Abcam, ab6558) was used.

### Statistical analysis of real-time PCR

All the statistical analyses conducted using SPSS 16 software and values were expressed as Mean ± SEM of three separate biological experiments. The ANOVA test was performed to compare the differences between eutopic, ectopic, and control samples. Statistical significance was defined as a *P* value of < 0.05.

## Results

### The expression analysis of *NF-YA*, *NF-YB* and *NF-YC* genes, and *ID* gene family

**Fig. 1:**
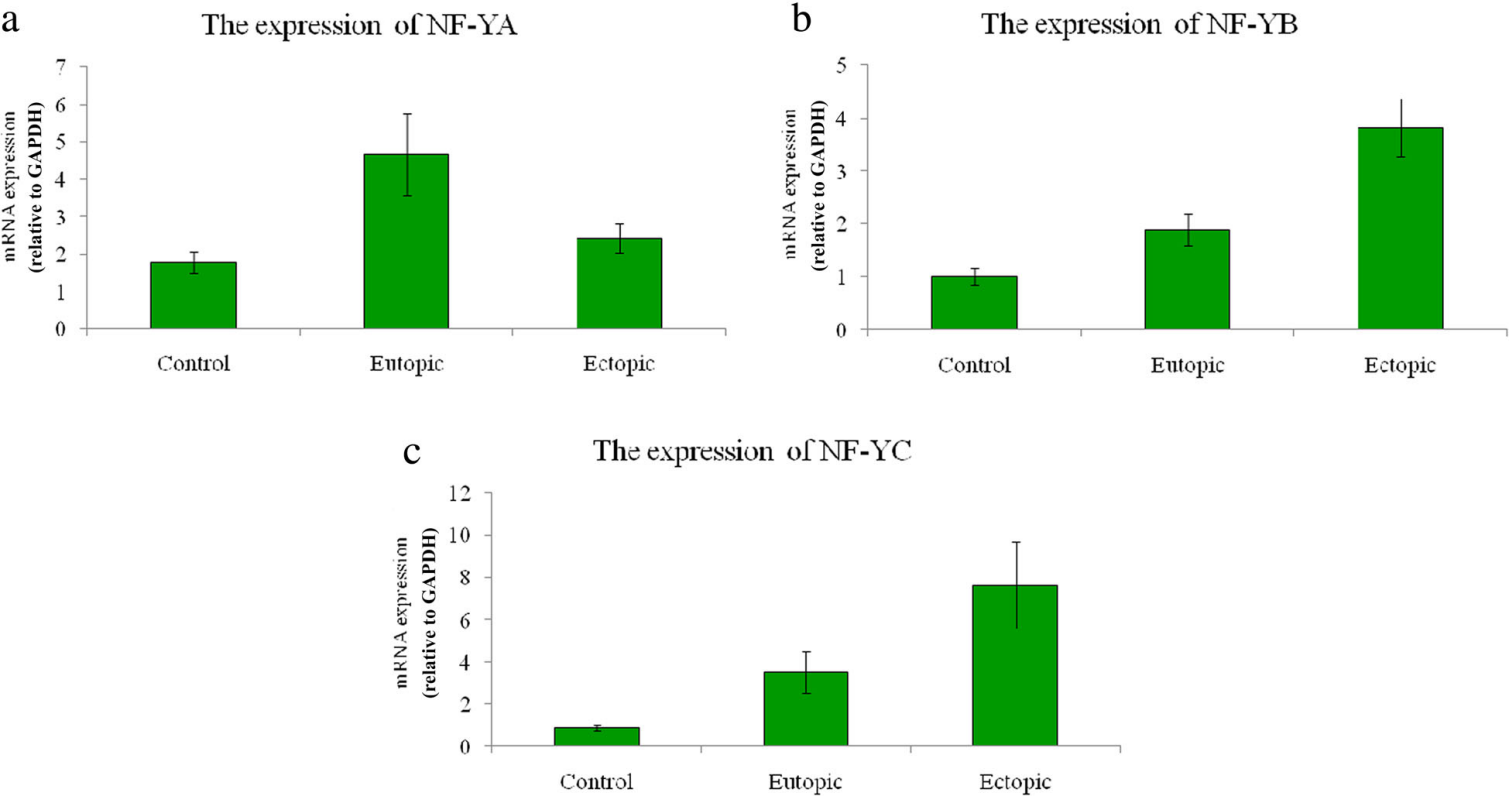
The expression profile of *NF-Y* subunits in endometriosis patients vs. control group in proliferative phase. Although the NF-YA overexpression in eutopic tissue samples (*n* = 20) is evident (*p* = 0.006), there is no remarkable change in its expression level in ectopic specimens (*n* = 20) compared to normal group (*n* = 20) (*p* = 0.730). The NF-YB and NF-YC were up-regulated in ectopic tissues (*p* = 0.000 and 0.002, *respectively*), while remained unchanged in eutopic samples (*p* = 0.277 and 0.397, *respectively*). (The measures are provided as mean ± SEM)

**Fig. 2:**
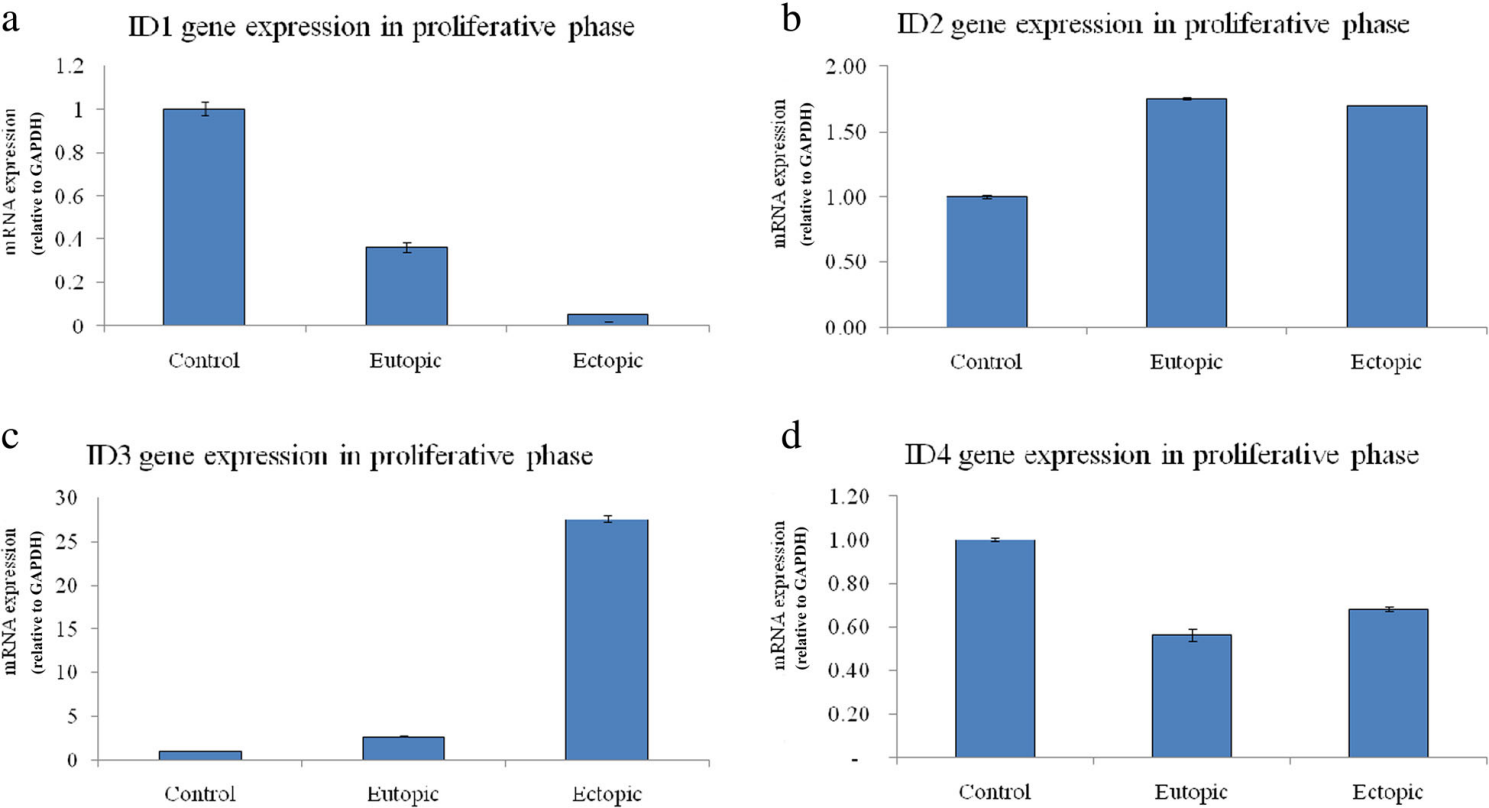
The expression profile of *ID* genes in endometriosis patients vs. control group in proliferative phase. The ID1 and ID4 expression levels were reduced in both ectopic (n = 20) (*p* = 0.001 and 0.015, *respectively*) and eutopic (*n* = 20) (*p* = 0.025 and 0.019, *respectively*) samples in comparison with control specimens (*n* = 20). On the other hand, ID2 and ID3 were overexpressed in ectopic (*p* = 0.0006 and 0.005, *respectively*) and eutopic tissues (*p* = 0.012 and 0.004, *respectively*). (The measures are presented as mean ± SEM)

To quantify the relative expression levels of *NF-Y* (*NF-YA*, *NF-YB*, and *NF-YC*) and *ID* (*ID1*–*ID4*) genes in ectopic, eutopic, and control groups, quantitative real-time PCR was performed (Figs. 1 and 2) (Table 2) during the proliferative phase. As illustrated in Fig. 1a, the expression level of *NF-YA* is increased in eutopic endometrium in comparison to control (3.39 ± 1.09 versus 1.77 ± 0.29) (*P* = 0.006) (Table 2). In addition, the expression of *NF-YA* in eutopic tissues was greater than the ectopic samples (2.41 ± 0.38) (*P* = 0.039). The expression level of *NF-YB* and *NF-YC* had no remarkable changes in eutopic tissue (1.88 ± 0.3 and 3.51 ± 0.97, *respectively*) in relation to control (1.01 ± 0.15 and 0.88 ± 0.13, *respectively*) (*P* = 0.277 and *P* = 0.397, *respectively*) (Table 2). On the other hand, their expression significantly increased in ectopic group (*NF-YB*: 3.81 ± 0.55, and *NF-YC*: 7.63 ± 2.05) during the proliferative phase compared to that of control (*P* = 0.000 and *P* = 0.002, *respectively*) (Table 2). *NF-YB* and *NF-YC* gene expression changes in the ectopic tissues were significant compared to eutopic tissues (Fig. 1b and c).

**Table 2:** Expression analysis of genes encoding NFY complex subunits and ID gene family members and the evaluation of NFY complex incorporation on *ID* genes upstream regions in normal and endometrial ectopic and eutopic tissue specimens

Tissue	Gene	qPCR	*P*-value	ChIP-seq	*P*-value
Eutopic	*NF-YA*	3.39 ± 1.09	0.006		
	*NF-YB*	1.88 ± 0.3	0.277		
	*NF-YC*	3.51 ± 0.97	0.397		
	*ID1*	0.36 ± 0.02	0.025	0.34 ± 0.045	0.195
	*ID2*	1.75 ± 0.01	0.012	0.45 ± 0.088	0.014
	*ID3*	2.67 ± 0.09	0.004	0.30 ± 0.037	0.025
	*ID4*	0.56 ± 0.03	0.019		
Ectopic	*NF-YA*	2.41 ± 0.38	0.730		
	*NF-YB*	3.81 ± 0.55	0.000		
	*NF-YC*	7.63 ± 2.05	0.002		
	*ID1*	0.05 ± 0.03	0.001	0.05 ± 0.016	0.005
	*ID2*	1.7 ± 0.00	0.0006	0.20 ± 0.076	0.799
	*ID3*	27.54 ± 0.4	0.005	0.58 ± 0.02	0.025
	*ID4*	0.68 ± 0.01	0.015		
Normal	*NF-YA*	1.77 ± 0.29			
	*NF-YB*	1.01 ± 0.15			
	*NF-YC*	0.88 ± 0.13			
	*ID1*	1 ± 0.03		0.25 ± 0.032	
	*ID2*	1 ± 0.01		0.14 ± 0.008	
	*ID3*	1 ± 0.03		0.19 ± 0.034	
	*ID4*	1 ± 0.01			

In proliferative phase, the expression level of *ID1*, *ID2*, *ID3*, and *ID4* genes in control, ectopic, and eutopic groups were assessed (Fig. 2a-c) (Table 2). The results indicated the down-regulation of *ID1* and *ID4* genes in both ectopic (*ID1*: 0.05 ± 0.03, and *ID4*: 0.68 ± 0.01) (*P* = 0.001, and *P* = 0.015) and eutopic (*ID1*: 0.36 ± 0.02, *ID4*: 0.56 ± 0.03) (*P* = 0.025, and *P* = 0.019) tissues in proliferative phase (Fig. 2a, d) (Table 2). However, the expression level of *ID2* and *ID3* was observed to be increased in ectopic (*ID2*: 1.7 ± 0.00, *ID3*:27.54 ± 0.4) (*P* = 0.0006, and *P* = 0.005) and eutopic (*ID2*: 1.75 ± 0.01, *ID3*: 2.67 ± 0.09) (*P* = 0.012, and *P* = 0.004) tissues (Fig. 2b, c) (Table 2).

### NF-Y incorporation on regulatory regions of *ID* genes in ectopic and eutopic tissues of women with endometriosis

To gain insight into NF-Y-mediated transcriptional regulation of *ID* genes in endometriosis, we investigated the occupancy of NF-Y complex on CCAAT regulatory region and/ or its complementary sequence in eutopic, ectopic, and normal tissues during the proliferative phase using ChIP analysis followed by real time PCR.

**Fig. 3:**
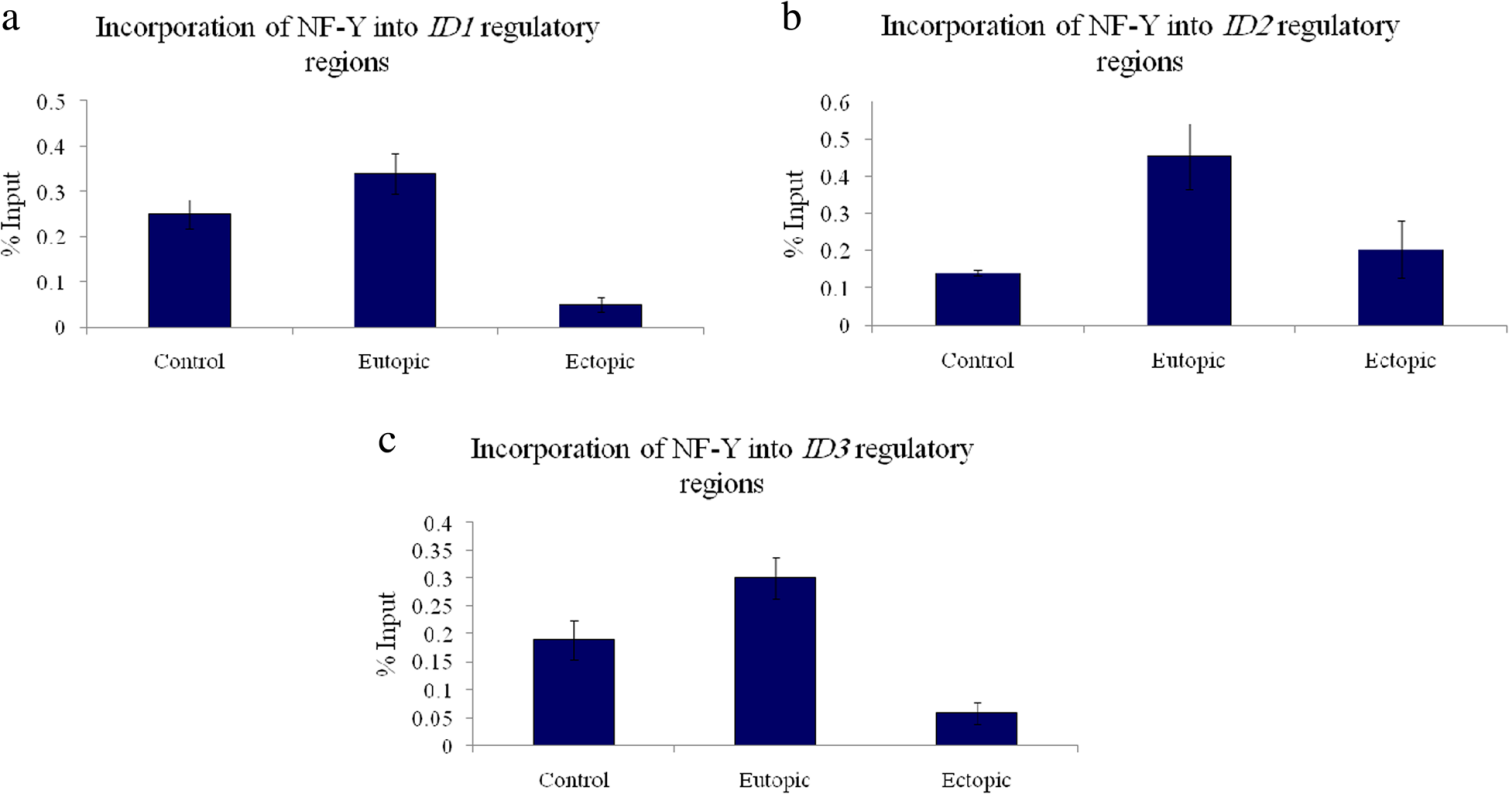
Incorporation of NF-Y complex on the regulatory regions of **a**: *ID1*, **b**: *ID2*, and **c**: *ID3* genes in endometriosis patients vs. control group in proliferative phase. The data shows statistically significant NFY complex enrichment on ID2 (*p* = 0.014) and ID3 (*p* = 0.025) promoters in eutopic group (*n* = 20), but notable reduction in NFY binding to ID1 (*p* = 0.005) and ID3 (*p* = 0.025) upstream regions in ectopic group (*n* = 20) in comparison with normal group (*n* = 20). (The measures represent mean ± SEM)

The ChIP analysis of the eutopic samples revealed significant enrichment of NF-Y on the CCAAT-containing regions of *ID2*, and *ID3* promoters in the proliferative phase (*ID2*: 0.45 ± 0.088, and *ID3*: 0.30 ± 0.037) in comparison with the normal samples (*ID2*: 0.14 ± 0.008, and *ID3*: 0.19 ± 0.034) (*P* = 0.014 and *P* = 0.025, *respectively*) (Fig. 3b, c) (Table 2). However, there was no remarkable difference in NF-Y binding level to the regulatory regions of *ID1*gene between eutopic (*ID1*: 0.34 ± 0.045) and control (*ID1*: 0.25 ± 0.032) tissues (*P* = 0.195) (Fig. 3a) (Table 2).

Comparing the level of NF-Y binding to the upstream regions of *ID* genes in ectopic and control specimens in proliferative phase, it was found that the binding level to the *ID1* and *ID3* CCAAT boxes was decreased in ectopic tissue samples (*ID1*: 0.05 ± 0.016, and *ID3*: 0.58 ± 0.02) (*P* = 0.005 and *P* = 0.025, *respectively*) (Fig. 3a, c) (Table 2). In ectopic group, a slight increment of binding to the *ID2* upstream regions (*ID2*: 0.20 ± 0.076) was observed which was not statistically significant (*P* = 0.799) (Fig. 3b) (Table 2).

Eventually, NF-Y incorporation on the *ID1*, *ID2* and *ID3* genes in eutopic group was remarkably greater than the ectopic group (*P* = 0.000, *P* = 0.045, and *P* = 0.000, *respectively*) (Fig. 3). However, the incorporation of NF-Y was not identified on *ID4* gene.

## Discussion

Endometriosis is a benign progressive disease with endometrial lesions leading to infertility in women of reproductive age [30]. According to the recent investigations, endometriosis is caused by several factors and the epigenetic plays a major role in development of the disease [10, 31, 32].

The NF-Y is one of the factors responsible for epigenetic changes. This factor facilitates the interaction between promoter and the enhancers by binding to the CCAAT BOX regulatory region through affecting the DNA condensation. This is a crucial process in the expression regulation of genes with this motif [13, 33]. Any changes in NF-Y (whether its expression or DNA binding properties) is regarded as a disease causing factor [13].

The current study revealed the up-regulation of *NF-YB* and *NF-YC* genes in ectopic tissue of endometriosis patients in the proliferative phase of the menstrual cycle which was significant in comparison to the control group. The *NF-YA* gene expression was remarkably increased in the endometrium of endometriosis patients (eutopic tissue) than that of the control group and the augmentation in this group was higher than the ectopic group.

Previous studies demonstrated that the cell growth and proliferation in endometriosis patients is increased in the proliferative phase [34]. It was also declared that some signaling pathways, related to the increased cell division and survival, are more activated in endometriotic cells of these patients [35]. Furthermore, the eutopic and ectopic endometriums have less differentiated cells which results in decreased levels of apoptosis in such tissues [35]. NF-Y regulates the balance between proliferation and differentiation states through the recruitment of RNA polymerase II and communication with transcription factors and the enzymes binding to the promoter regions of cell cycle-regulated genes [13, 36, 37].

In present study, the binding level of NF-Y factor to the regulatory regions of *ID1*, *ID2*, and *ID3* genes in endometrium of the control and patient groups was compared in the proliferative phase in order to epigenetic assessments.

The *ID* family genes (*ID1*, *ID2*, *ID3*, and *ID4*) are major regulators of two important cell processes: the proliferation and differentiation. These genes play important roles in signaling pathways, cell fate determination, cell death, tumorigenesis, cell cycle and others as a result of their ability to restrain the differentiation and stimulate the proliferation [38–40]. An interesting feature of *ID* family genes (which led us to select them for the current research) is the existence of CCAAT regulatory region or its complementary sequence (ATTGG) in their promoter which affects the regulated binding of NF-Y to this region. It is worth to mention that among all the members of ID gene family, the *ID4* is deficient in the presence of CCAAT sequence in its upstream region to be occupied by the NFY complex. The binding of NF-Y protein to the *ID* gene promoter implies that such connection is necessary for transcription initiation in the cell division process [33].

Regarding the results from quantitative comparison of *ID1*, *ID2*, *ID3*, and *ID4* gene expression between the control and endometriosis groups in the proliferative phase, the *ID2* and *ID3* gene expression in both ectopic and eutopic tissues were significantly higher than the control group and the expression level of *ID1* and *ID4* genes was decreased in the ectopic and also eutopic tissue.

Among the factors affecting the expression of *ID* gene family, c-Myc is a reasonable candidate for being responsible for *ID* gene family expression changes in endometriosis patients. C-Myc, a multifunctional nuclear phosphoprotein, participates in the cell cycle and apoptosis processes as a transcription factor regulating the expression of certain genes [41]. C-Myc contains a binding site for *ID* genes’ promoter which has more affinity for *ID2* promoter than others. However, it was clarified that *ID3* promoter is also a target for this protein in recent years [42, 43]. The previous investigations indicated that c-Myc factor increases the expression of these genes. The studies revealed that the expression of c-Myc as a regulating factor for cell cycle is increased in the eutopic and ectopic tissues of endometriosis patients in the proliferative phase in comparison to normal people. The elevated level of c-Myc expression in endometriosis patients seems to be related to the estrogen level increment and also TGF-β expression reduction [44]. Furthermore, it has formerly been disclosed that in endometrial tissues, there is an elevated level of *ID2* gene expression negatively affected by increased levels of TGF-β [45]. This expression pattern exhibits conformity to the expression of *ID2* and *ID3* in current study. Regarding the similar expression patterns, it can be interpreted that c-Myc family is responsible for *ID2* and *ID3* up-regulation in eutopic and ectopic tissues in the proliferative phase.

Moreover, the helix-loop-helix (HLH) domain is conserved in all the members of *ID* family but the C- and N-terminal regions are different which provides the possibility of interaction between *ID* genes and different factors resulting in various consequences [46]. The hematopoietic stem cell studies indicated that each member of *ID* family participates in a certain phase of hematopoietic cell evolution. For example, once the cell is stimulated by inducing cytokines like Interleukine-3, Interleukine-6, and Erythropoietin, the *ID1* gene is over-expressed suggesting the role *ID1* plays in proliferation and pluripotency maintenance of hematopoietic stem cells. *ID1* gene also participates in cell fate determination in T cell or NK cell differentiation pathway but the expression of *ID2* is not notably increased in this phase and is significantly elevated in final stages (granulocyte differentiation) instead [47].

The above mentioned differences also lead to various *ID* family activities in different cells and tissues. For example, the over-expression of *ID1* gene in fetus cerebral cortex results in neural cell proliferation and *ID4* gene is necessary for mammary gland development [48–50].

The expression of *ID1*, *ID2*, and at a lesser amount the *ID3* genes were also investigated in several studies including prostate cancer researches and the relationship between their over-expression and cancer incidence was clearly perceived. On the other hand, some studies declared that the reduction of *ID4* gene expression is responsible for prostate cancer development. Other studies clarified that the *ID4* gene expression in normal breast and stomach tissues is highly elevated in comparison to cancerous tissues denoting the tumor suppressive role for this protein [51, 52].

The binding level of NF-Y protein complex to the promoter regions of *ID1*, *ID2*, and *ID3* genes was displayed through ChIP-Real-Time-PCR and according to the results; it was considerably increased in eutopic group of endometriosis patients in proliferative phase.

The function of NF-Y transcription factor is regulated by post-translational molecular mechanisms. The regulation of *NF-Y* is accomplished at protein level and through the NF-YA subunit [16, 53, 54]. The protein level of NF-Y is increased and/ or decreased depending on the cell status. It shows that the binding level of NF-Y protein fluctuates in cells and the regulation through NF-YA subunit prevents the whole complex from binding to the DNA.

The results indicated that the *NF-YA* is significantly over-expressed in eutopic tissue of endometriosis patients and the binding level of NF-Y complex to the promoter regions of *ID* genes in eutopic tissue is elevated as well. This is significant as the DNA binding of NF-Y complex is accomplished through NF-YA subunit and it is the only subunit possessing the DNA binding domain which without this domain the complex is not able to bind the CCAAT motif [55].

Comparing the binding level of NF-Y complex to the promoter regions of *ID1*, *ID2*, and *ID3* genes with quantitative data from expression of *ID1*, *ID2*, and *ID3* genes in control and endometriosis groups in proliferative phase, it was revealed that the level of NF-Y complex binding to the *ID2* promoter region in endometrium of endometriosis women (the eutopoic tissue) in proliferative phase of the menstrual cycle is augmented in comparison with the control group which is accompanied by over-expression of this gene in the endometrium and endometrial lesions of endometriosis patients in proliferative phase.

Moreover, a reduction in binding level of NF-Y to the *ID1* gene promoter was observed in endometrial lesions (ectopic tissue) in proliferative phase compared to the control group.

In contrast, the NF-Y binding to the *ID3* promoter region in the endometrial lesions (ectopic tissue) in proliferative phase is significantly decreased compared to the control group which is contradictory to *ID3* gene expression data in the proliferative phase.

The studies demonstrated that CCAAT box sequence (the sequence which NF-Y binds to) is one of the most common elements in the upstream of eukaryotic promoters. In addition to the CCAAT box motif, there are other conserved regions in class II eukaryotic promoters including GC-box and TATA-box [56]. Likewise, in addition to NF-Y protein, there are other proteins comprising HSP-CBF, Y-BOX FACTORS, CTF/NF-1, and C/EBF to bind to this site of DNA [55].

Therefore, the involvement of other conserved regulatory regions and/ or other regulatory elements in the process of *ID3* gene expression regulation in endometrial lesions (ectopic tissue) of endometriosis patients have a great likelihood. It was also determined that the genes without TATA box are more dependent to their CAT box region [12, 16].

The *ID2* gene has two CAT box regions and no TATA box, while the *ID1* and *ID3* genes have only one CAT box [33]. As the TATA box lacking genes are intensively dependent to their CAT box region, the role of NF-Y protein in *ID2* gene regulation and epigenetic in endometriosis patients is more significant than its binding to the *ID1* and *ID3* genes.

## Conclusion

The altered levels of *NF-YA*, *NF-YB*, and *NF-YC* expression resulted in some changes in the expression of *ID* gene family in both ectopic and eutopic tissues of endometriosis patients in proliferative phase. Furthermore, the incorporation of NF-Y complex on CCAAT box region of *ID1*, *ID2*, and *ID3* promoters was highly enhanced in endometriosis patients. Thus, it can be suggested that NF-Y transcription factor has regulatory role on *ID* gene family through CCAAT box region and is responsible for epigenetic changes in endometrial tissues of endometriosis patients.

However, more investigations are required to clarify the different underlying molecular regulatory mechanisms in eutopic and ectopic endometriums in endometriosis patients which may open novel avenues in understanding of endometriosis pathophysiology and give rise to novel therapeutic strategies.

## Abbreviations

bHLH: Basic helix-loop-helix
c DNA: Complementary DNA
ChIP: Chromatin immunoprecipitation
DEPEC: Diethylpyrocarbonate
HLH: Helix-loop-helix
*ID*: Inhibitor of differentiation
NF-Y: Nuclear transcription factor Y
RT: Reverse transcription
